# Mono-PEGylated thermostable *Bacillus caldovelox* arginase mutant (BCA-M-PEG20) induces apoptosis, autophagy, cell cycle arrest and growth inhibition in gastric cancer cells

**DOI:** 10.1007/s10637-022-01265-z

**Published:** 2022-07-20

**Authors:** Sai-Fung Chung, Suet-Ying Tam, Chi-Fai Kim, Hiu-Chi Chong, Leo Man-Yuen Lee, Yun-Chung Leung

**Affiliations:** Department of Applied Biology and Chemical Technology, Lo Ka Chung Research Centre for Natural Anti-Cancer Drug Development and State Key Laboratory of Chemical Biology and Drug Discovery, The Hong Kong Polytechnic University, Hung Hom, Kowloon, Hong Kong, China

**Keywords:** BCA-M-PEG20, Apoptosis, Autophagy, Cell cycle arrest Cytostatic

## Abstract

Gastric cancer is one of the most common malignant solid tumors in the world, especially in Asia with high mortality due to a lack of effective treatment. The potential usage of the newly constructed arginine-depleting enzyme—mono-PEGylated *Bacillus caldovelox* arginase mutant (BCA-M-PEG20), an effective drug against multiple cancer cell lines such as cervical and lung cancers, for the treatment of gastric cancer was demonstrated. Our results indicated that BCA-M-PEG20 significantly inhibited argininosuccinate synthetase (ASS)-positive gastric cancer cells, MKN-45 and BGC-823, while another arginine-depleting enzyme, arginine deiminase (ADI, currently under Phase III clinical trial), failed to suppress the growth of gastric cancer cells. In vitro studies demonstrated that BCA-M-PEG20 inhibited MKN-45 cells by inducing autophagy and cell cycle arrest at the S phase under 0.58 U/mL (IC_50_ values). Significant caspase-dependent apoptosis was induced in MKN-45 after the treatment with 2.32 U/mL of BCA-M-PEG20. In vivo studies showed that administrations of BCA-M-PEG20 at 250 U/mouse twice per week significantly suppressed about 50% of tumor growth in the MKN-45 gastric cancer xenograft model. Taken together, BCA-M-PEG20 demonstrated a superior potential to be an anti-gastric cancer drug.

**Supplementary information:**

The online version contains supplementary material available at 10.1007/s10637-022-01265-z.

## Introduction

Gastric cancer (GC) is one of the leading cancers worldwide with high mortality rates [1–3]. There are only limited curative therapeutic options to treat GC, with surgery and chemotherapy being the most promising treatments. However, these traditional treatments have various adverse side effects and the five-year overall survival rates of patients are extremely low [1–3]. To improve therapeutic outcomes, targeted therapy and immunotherapy have been launched to specifically interfere with the signaling pathways of gastric cancer cells to stop them from growing and dividing. The most common targeted pathways in GC are human epidermal growth factor receptor 2, programmed cell death protein and vascular endothelial growth factor [1, 3, 4]. Although the Food and Drug Administration (FDA) has approved different therapeutic antibodies, such as trastuzumab, ramucirumab and pembrolizumab, as first- and second-line treatments for GC [2, 3], their efficacies are poor on gastric cancer since only a small portion of patients are HER2 (~ 20%), VEGF and PD-1/PD-L1 positive [4, 5]. Therefore, an alternative way to treat GC is urgently needed.

Arginine depletion therapy is a bright and safe way to starve cancer cells by depriving arginine, a semi-essential amino acid, in cancer cells. Most cancer cells are arginine-auxotrophic, meaning they lack arginosuccinate synthetase (ASS) or ornithine transcarboxylase (OTC) expression in the urea cycle to recycle arginine; thus, arginine depletion therapy can be used to treat a broad spectrum of cancer cells, including melanoma, colorectal, lung, ovarian, and hepatocellular carcinoma [6–15]. Under arginine scarcity, cancer cells experience cell cycle arrest, autophagy, and subsequently undergo caspase-dependent apoptosis, leading to cytotoxicity [11, 13, 15–19].

Arginine deiminase (ADI) is a promising drug for arginine depletion therapy. It is an arginine-depleting enzyme with bacterial origin and has been developed in a random PEGylated form (ADI-PEG-20) to reduce its immunogenicity and prolong its half-life [20, 21]. Its PEGylated form has exhibited positive therapeutic outcomes in treating cancers and has been applied in different clinical trials, mainly for treating melanoma and HCC [22–26]. However, one of the limitations of this drug is that its efficacy relies on the expression of ASS in cancer cells [12, 27–29]. When cancer cells are ASS-positive, arginine can be re-generated from citrulline, resulting in the failure of arginine deprivation. The majority of cancer cells are ASS-positive, therefore they cannot be cured by ADI-PEG20. Additionally, the production of ADI-PEG20 involves complicated unfolding and refolding procedures which result in non-homogeneous products with batch-to-batch variations [20, 30].

As far as we know, arginine depletion therapy has not yet been investigated for the treatment of GC. In this study, we explored the potential of arginine depletion therapy for GC by using a mono-PEGylated mutated *Bacillus caldovelox* arginase (BCA-M-PEG20) [9]. It is an alternative arginine-depleting enzyme with promising anti-cancer effects on lung and cervical cancers already demonstrated [9, 16]. Our study showed that BCA-M-PEG20 was superior to ADI/ADI-PEG20 in inhibiting ASS-positive but OTC-negative GC cells. Unlike other arginine depletion drugs that have cytotoxicity on cancer cells, our BCA-M-PEG20 was proved to play an excellent cytostatic role on GC cell lines. Its anti-proliferation effect was mainly attributed to a significant cell cycle arrest at S phase and the induction of autophagy associated with a minor caspase-dependent apoptosis. Furthermore, with an injection of BCA-M-PEG20 (250 U/mouse) twice a week, about 50% tumor suppression in mice was observed while there were no dramatic changes in the body weight. BCA-M-PEG20 exhibited a comparable inhibitory effect to the positive control, 5-fluorouracil, indicating that it is a promising drug candidate for treating GC.

## Materials and methods

### Materials

MTT (3-(4,5-dimethylthiazol-2-yl)-2,5-diphenyltetrazolium bromide) reagent was purchased from Invitrogen Life Technologies (San Diego, CA, USA). RIPA Lysis Buffer and Pierce BCA Protein Assay Kit were obtained from Thermo Fisher Scientific (HK). Twenty kDa linear PEG conjugated with maleimide functional group was purchased from NOF America Corporation (White Plains, NY).

### Production of BCA-M-PEG20 and ADI

The production protocols were the same as those in our previous studies [9, 30].

### Cell proliferation assay

Cytotoxicity of BCA-M-PEG20 against MKN-45 and BGC-823 cells was determined by MTT assay as described previously [9, 16]. Then, the results of the optical density at a wavelength of 570 nm were detected by a Varioskan LUX Multimode Microplate Reader.

### Western blot analysis

MKN-45 cancer cells with or without BCA-M-PEG20 treatments at different concentrations and times were harvested during the log-phase. Cells and a small piece of mouse liver (as ASS- and OTC-positive control) were lysed with RIPA lysis buffer on ice for 15 min. Then, the extracted cellular proteins were obtained by centrifugation at 15,000 rpm for 5 min. Pierce™ BCA Protein Assay was used to determine the concentration of the extracted cellular proteins. An equal amount of total protein per lane was loaded and separated by electrophoresis separation, then transferred to Immobilon-P polyvinylidene fluoride membranes. The membranes were blocked with 5% blotting-grade blocker with Tris-buffered saline (TBST, 0.1% Tween-20, 100 mM Tris–HCl, pH 7.5, 0.9% NaCl) at room temperature for 1 h. Then the membranes were incubated with a specific primary antibody at 4 °C overnight. After incubation, the membranes were washed by TBST and incubated with secondary antibodies at room temperature for 1 h. Excess secondary antibodies were removed by washing with TBST. The detection of specific protein was performed by using the Immobilon Western Chemiluminescent HRP Substrate (Millipore Corporation, Billerica, MA, USA). For the primary antibodies, rabbit anti-ASS, rabbit anti-OTC, rabbit anti-PARP, rabbit anti-cleaved caspase-3, and rabbit anti-beta-actin (1:1000, Cell Signaling Technology, Danvers, MA, USA) were used. HRP-conjugated goat anti-rabbit secondary antibody (1:10,000, Cell Signaling Technology, Danvers, MA, USA) was applied to determine all primary antibodies. ImageJ software (National Institutes of Health) was adopted to determine the protein signal intensities. A more comprehensive western blot results are shown in supplementary materials (Figs. S1-S3 and S5).

### Apoptosis assay

To detect apoptotic cells, Annexin V-FITC/PI Apoptosis Detection Kit (BD Biosciences, San Diego, CA, USA) was applied. A density of approximately 0.4 × 10^6^ MKN-45 cells was seeded into a T25 flask and incubated overnight prior to BCA-M-PEG20 treatments. Cells treated with or without BCA-M-PEG20 at various concentrations and time points were harvested and washed by PBS twice, followed by resuspension in 1X assay buffer and incubation with Annexin V-FITC and/or PI for about 15 min in the dark. The stained cancer cells were then subjected to BD Accuri C6 Flow Cytometer within 1 h.

### Cell cycle analysis

MKN-45 cells were seeded, treated, and harvested as in apoptosis assay but without the collecting of dead cells, followed by fixation in 60% ethanol at 4 °C overnight. Then, the fixed cells were washed by PBS twice, filtered via a 60 µm nylon mesh and incubated with PI/RNase staining buffer at room temperature for at least 1 h. Finally, fluorescence-activated cell sorting analyses were carried out on the stained cells and data from each sample were collected from at least 10,000 cells. ModFit LT 3.1 (Verity Software House, Topsham, ME, USA) was used to determine the cell cycle distribution analyses.

### Autophagy detection by confocal microscope

To monitor the autophagy of live MKN-45 cells induced by BCA-M-PEG20, CYTO-ID® Autophagy detection kit was used (Enzo Life Sciences, Farmingdale, NY, USA). About 6000 MKN-45 cancer cells were seeded into confocal plates per well and incubated with or without BCA-M-PEG20 (0.58 µg/mL) for different durations. A positive control, Rapamycin (500 nM), was used in this assay. Then, cancer cells were stained with cyto-ID dye and Hoechst 33,342 following the instructions of the manufacturer and detected by a Leica TCS SPE confocal microscope.

### Mouse xenograft model of gastric cancer treated by BCA-M-PEG20

For the preparation of MKN-45 tumor xenograft, 1 × 10^6^ MKN-45 cells were injected into the flank of BALB/c nude mice subcutaneously. When the size of tumors reached 1.5–2.0 cm in diameter, they were excised, cut into tumor fragments, and implanted into the nude mice. Then, the tumor volume was measured closely. Once a stable growth of tumors was maintained, the mice were randomly divided into 3 groups. Ten mice with an average tumor volume of about 400 mm^3^ were assigned into one group. BCA-M-PEG20 (250 U/mouse) and 5-fluorouracil (5-FU, 10 mg/kg) were administrated twice per week and once per week to nude mice bearing tumor xenograft through i.p. injection, respectively. The control group was injected with PBS as vehicle control. Tumor dimensions were determined in situ regularly throughout the treatment period using a digital caliper and the tumor volume was estimated by the Eq. 0.5 × length x (width)^2^. The bodyweight of the tumor bearing mice was monitored every week throughout treatment. Lastly, the mice were sacrificed by cervical dislocation and the tumors were obtained for actual tumor weight measurement.

## Results

### BCA-M-PEG20 inhibited the growth of MKN-45 and BGC-823 cancer cells

**Fig. 1:**
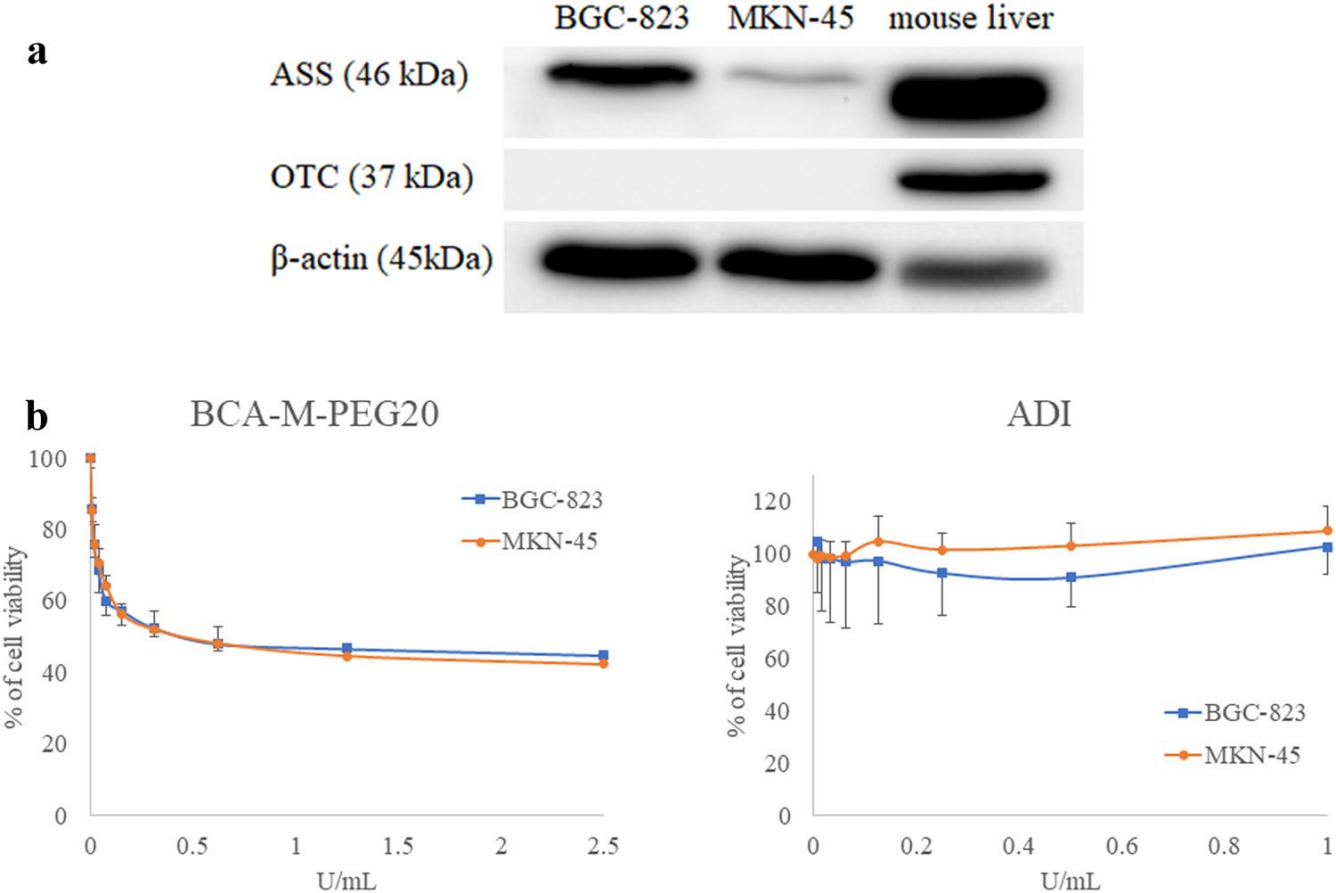
Growth inhibitory effects on gastric cancer cell lines after the treatment of BCA-M-PEG20. **a** Protein expressions of ASS and OTC were measured by western blot analysis in MKN-45 and BGC-823. Mouse liver served as a positive control. **b** Cell proliferation assay for BCA-M-PEG20 and ADI on BGC-823 and MKN-45 cell lines were determined by MTT assay. Three independent experiments were performed in triplicate (each n = 3)

Our previous studies and other researchers reported that cancer cells that were either OTC- or ASS-negative were sensitive to arginine depletion therapy induced by human arginase or *Bacillus caldovelox* arginase [16, 17]. As shown in Fig. 1a, MKN-45 and BGC-823 cancer cells were OTC-negative but ASS-positive, and mouse liver was used as a positive control. Moreover, our results indicated that BCA-M-PEG20 significantly suppressed the growth of ASS-positive but OTC-negative MKN-45 and BGC-823 cancer cell lines (Fig. 1b and Table 1). The IC_50_ values of BCA-M-PEG20 on MKN-45 and BGC-823 were 0.58 ± 0.11 and 0.63 ± 21 U/mL, respectively. In contrast, both MKN-45 and BGC-823 were resistant to arginine deiminase (ADI) treatment due to the expression of ASS gene, even when the drug concentration reached 1 U/mL, which was about 400-fold higher than the dosage used on two sensitive cervical cancer cell lines, C33-A and SiHa [16] (Table 1).

**Table 1:** IC_50_ values of BGC-823 and MKN-45 cells treated with BCA-M-PEG20 and ADI

	IC_50_ (U/mL)				References
	BCA-M-PEG20	ADI	ASS	OTC	
BGC-823	0.63 ± 0.21	> 0.125	+	-	Current study
MKN-45	0.58 ± 0.11	> 0.125	+	-	Current study
	BCA-M	ADI			
C-33A	0.19	0.00147	-	-	[16]
SiHa	0.31	0.00299	-	-	[16]

### Analysis of apoptosis induced by BCA-M-PEG20

**Fig. 2:**
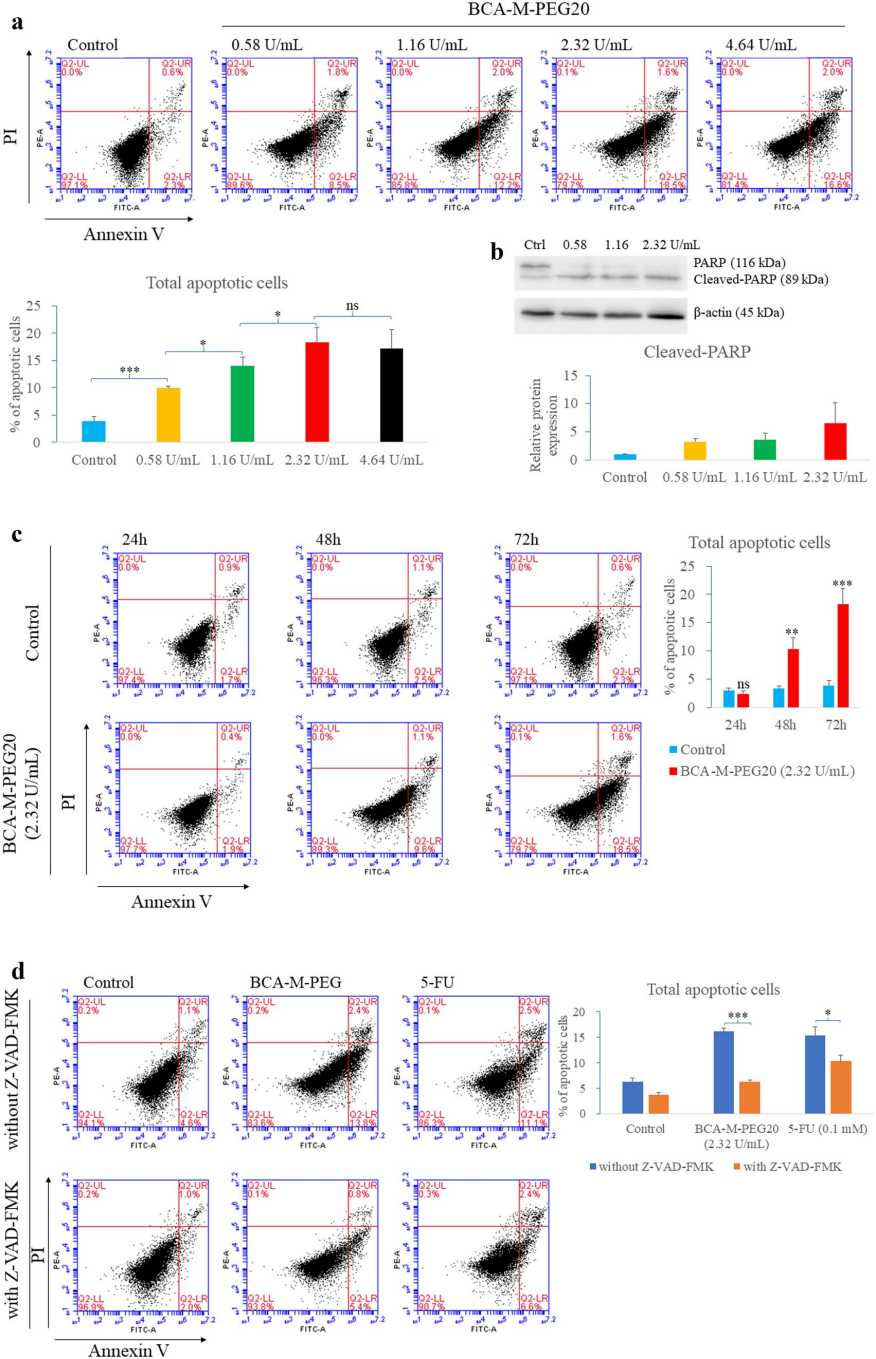
BCA-M-PEG20 induced apoptosis in MKN-45 cells in a dose-dependent manner after 72 h of incubation as shown by **a** flow cytometry with Annexin V-FITC and propidium iodide staining, and **b** western blot analysis of cleaved-PARP level. The percentages of apoptotic cells are presented in the bar charts and band intensity was quantified using the ImageJ software. **c** BCA-M-PEG20 at a dosage 2.32 U/mL induced apoptosis in MKN-45 cells in a time-dependent manner. **d** MKN-45 cells were treated with 2.32 U/mL BCA-M-PEG20 and 0.1 mM 5-FU (positive control) with or without the caspase inhibitor z-VAD-FMK for 72 h. The results are shown as mean ± S.D and analyzed by Student’s t-test (n = 3, ^*^p < 0.05, ^**^p < 0.01, ^***^p < 0.001)

After the treatment of BCA-M-PEG20 at different doses and durations on MKN-45 cells, flow cytometry results showed that the apoptotic cells increased in both dose- and time-dependent manner. Notable apoptotic cells from 9.97 to 18.32% were detected at 72 h incubation with 0.58 to 2.32 U/mL of BCA-M-PEG20 treatment (Fig. 2a) but not with 0.29 U/mL dosage (Fig. S4a). Furthermore, western blot analysis showed the amount of cleaved PARP, an apoptosis marker, increased at 72 h induced by BCA-M-PEG20 (Fig. 2b), an observation that was in agreement with the flow cytometry results. Moreover, as shown in Fig. 2c, a time-dependent increase in the percentage of apoptotic cells in MKN-45 with 2.32 U/mL of BCA-M-PEG20 was observed from 24 to 72 h incubation. To elucidate whether arginine depletion in MKN-45 cells could induce caspase-dependent or caspase-independent apoptosis pathways, z-VAD-FMK, a caspase inhibitor, was used in combination with BCA-M-PEG20, while the combination of z-VAD-FMK with 5-FU served as a positive control. z-VAD-FMK could partially rescue MKN-45 cells under BCA-M-PEG20 and 5-FU treatments (Fig. 2d). Moreover, cleaved caspase-3 was detected after 72 h treatment of 2.32 U/mL of BCA-M-PEG20 (Fig. S3). These results supported that BCA-M-PEG20 elicited caspase-dependent apoptosis in MKN-45 cells.

### Analysis of cell cycle distribution induced by BCA-M-PEG20

**Fig. 3:**
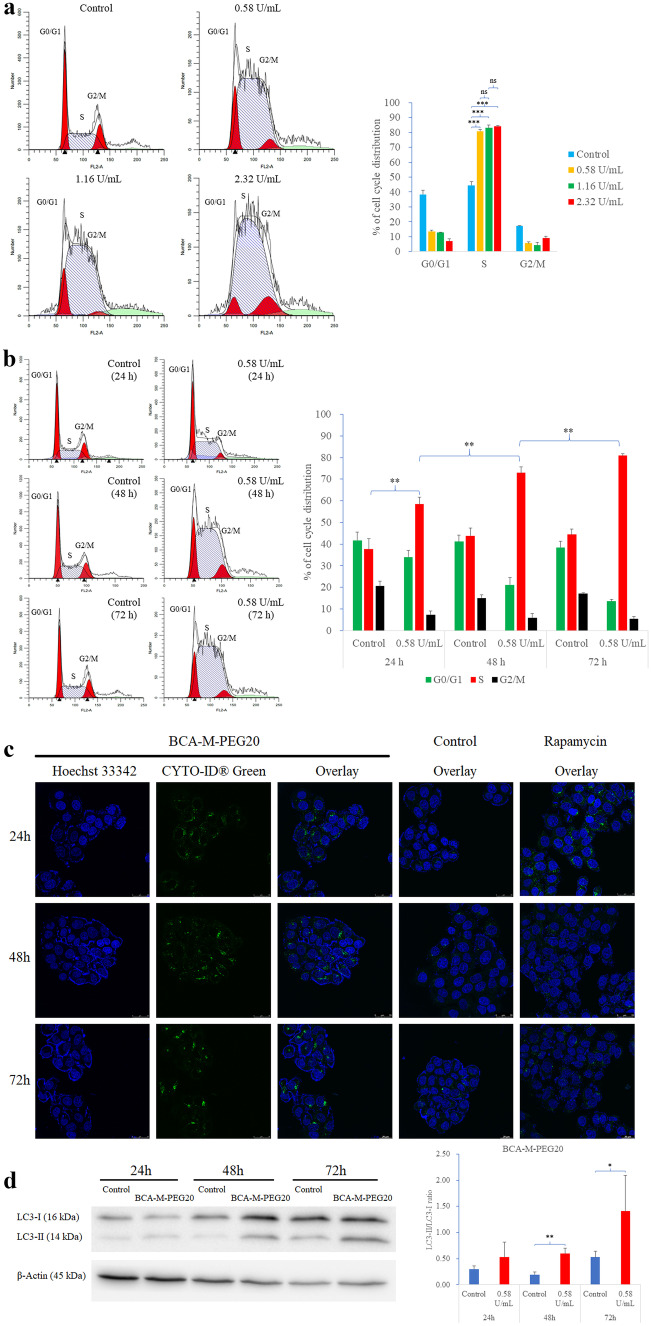
BCA-M-PEG20 induced cell cycle arrest and autophagy in MKN-45 cells. **a** BCA-M-PEG20 induced S phase arrest in MKN-45 cells after 72 h of incubation as shown by flow cytometry with propidium iodide staining. The percentages of cell distribution are presented in the bar charts. **b** BCA-M-PEG20 with 0.58 U/mL resulted in S phase arrest in MKN-45 cells in a time-dependent manner. **c** Green punctate signals represented autophagosome formation in MKN-45 after 24–72 h of BCA-M-PEG20 (0.58 U/mL) treatment. **d** An increase in the ratio of LC3-II/LC3-I was detected after the treatment of 0.58 U/mL of BCA-M-PEG20. The results are shown as mean ± S.D and analyzed by Student’s t-test (n = 3, ^*^p < 0.05, ^**^p < 0.01, ***p<0.001)

After the treatment of BCA-M-PEG20 at different doses and durations in MKN-45 cells, flow cytometry with propidium iodide staining was used to determine the cell cycle distribution. Our results showed that 0.58 to 2.32 U/mL of BCA-M-PEG20 treatment elicited a dramatic increase in the percentage of MKN-45 cells in the S phase and a decrease in both G0/G1 and G2/M phases after 72 h of incubation (Fig. 3a). In addition, the treatment of 0.58 U/mL of BCA-M-PEG20 resulted in a statistically progressive increase in the S phase population of MKN-45 cells from 24 to 72 h with a concomitant decrease in both G0/G1 and G2/M phases (Fig. 3b). Even BCA-M-PEG20 with half of the IC_50_ value (0.29 U/mL) still induced a portion of MKN-45 cells in the S phase (Fig. S4b), suggesting that the S phase arrest might be a dominant anti-cancer pathway.

### BCA-M-PEG20 induced autophagy in MKN-45 cells

Our results showed that the positive control 500 nM rapamycin induced LC3-II puncta formation on MKN-45 cells in 24 h but the effect did not last to 48 and 72 h (Fig. 3c). Interestingly, MKN-45 cells treated with 0.58 U/mL of BCA-M-PEG20 displayed a notable increase in LC3-II puncta formation together with a longer duration of up to 72 h than rapamycin, suggesting that BCA-M-PEG20 could be a potent and long-lasting autophagy inducer. Western blot analysis (Fig. 3d) showed that BCA-M-PEG20 could significantly increase the LC3-II/LC3-I ratio after 48 and 72 h of treatment, a result that was consistent with the observation from the confocal microscope.

### Analysis of BCA-M-PEG20 efficacy on MKN-45 xenograft mouse model

**Fig. 4:**
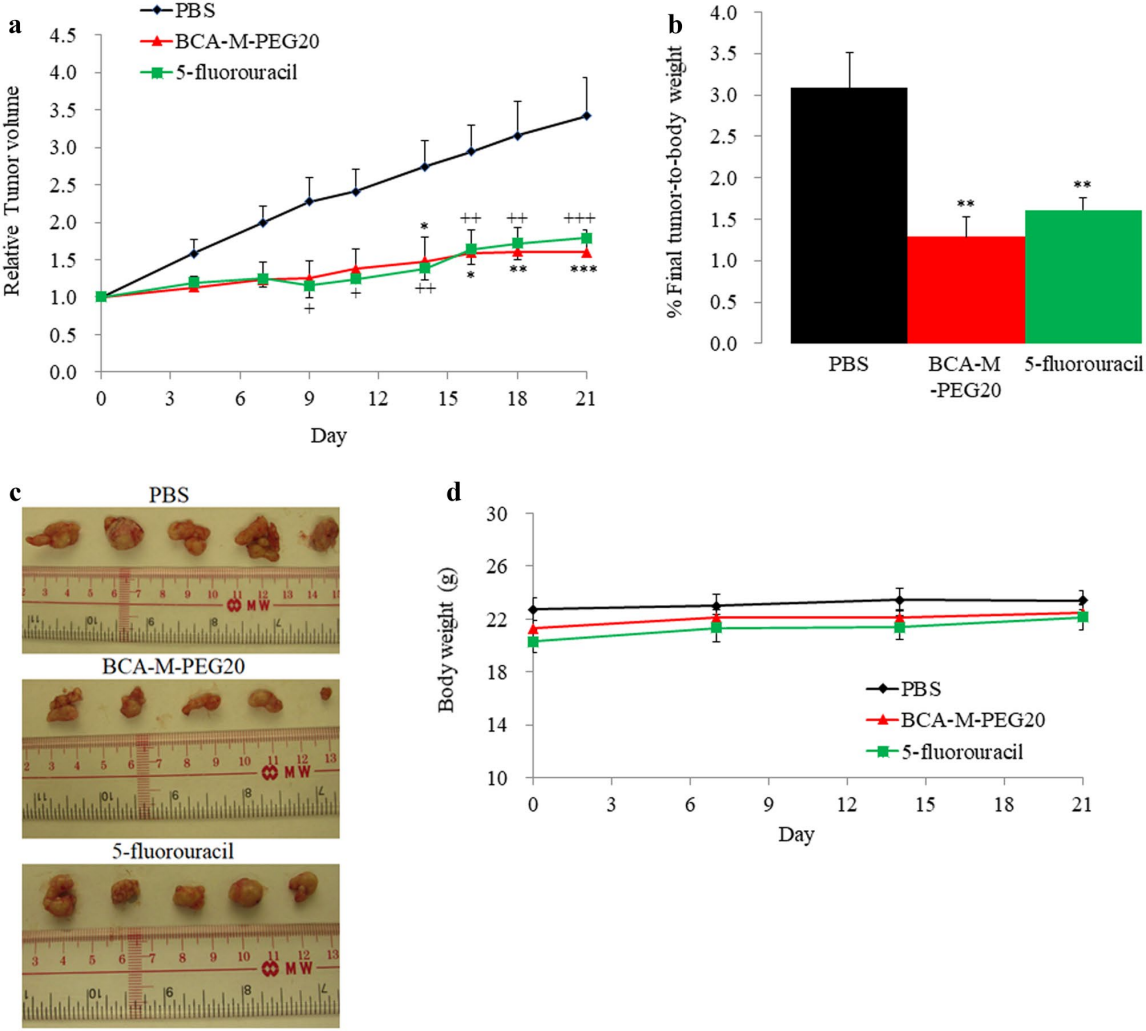
BCA-M-PEG20 and 5-fluorouracil (positive control) showed notable tumor inhibition in term of **a** Relative tumor volume and **b** % final tumor-to-body weight. **c** Macroscopic appearance of tumors dissected from PBS, BCA-M-PEG20 and 5-fluorouracil treated mice after the experiment (n = 10). **d** The body weight of each mouse in the three groups was measured weekly. The results are shown as mean ± SEM. through Two-way ANOVA with Bonferroni correction. ^*^P < 0.05, ^**^P < 0.01 and ^***^P < 0.001

To determine the drug efficacies of BCA-M-PEG20 and 5-fluorouracil, MKN-45 xenograft mouse models were constructed. The control group with PBS injection showed progressive tumor growth, whereas the BCA-M-PEG20 and 5-fluorouracil (positive control) groups displayed significant tumor suppression (about 50% tumor suppression) as shown in Fig. 4a, b and c. No notable bodyweight losses were observed in any groups during the experiment as shown in Fig. 4d.

### Discussion

GC is one of the toughest cancers with poor overall survival rates due to a lack of effective targeted therapy, and its treatment highly relies on invasive therapeutic approaches such as surgery and chemotherapy. Thus, it is urgent to develop a safe and promising alternative way to treat GC.

Arginine depletion therapy is a relatively new, safe, and promising anti-cancer strategy for treating various cancer cell lines such as lung [9, 31], liver [8, 32], laryngeal [33] and colorectal cancers [6]. The PEGylated form of ADI (ADI-PEG20) has shown satisfactory therapeutic results in treating liver cancer and melanoma and has been applied in clinical trials [22, 24], but its therapeutic efficacy is associated with the expression of ASS, it becomes ineffective when the cancer cells are ASS-positive or when the expression of ASS gene is up-regulated during the ADI-PEG20 treatment. Unlike ADI-PEG20, our novel BCA-M-PEG20 could treat not only ASS-negative but also OTC-negative cancer cell lines. Therefore, BCA-M-PEG20 was used to investigate the drug efficacy and cellular pathways on ASS-positive but OTC-negative GC cell lines in vitro and in vivo.

This study is the first report that shows GC cell lines are sensitive to arginine depletion therapy. Our results demonstrated that both MKN-45 and BGC-823 cells were ASS-positive but OTC-negative, meaning they were ADI/ADI-PEG20-resistant but BCA-M-PEG20-sensitive (Fig. 1). To evaluate the effect of BCA-M-PEG20 on the MKN-45 cell line, its IC_50_ dosage (0.58 U/mL) was chosen for the investigation of apoptosis, cell cycle, arrest and autophagy cellular pathways. Intriguingly, the incubation of 0.58 U/mL of BCA-M-PEG20 for 72 h not only significantly elicited approximately 40% increase in S phase (Fig. 3b), but also demonstrated obvious autophagosome and LC3-II formations (Fig. 3c). The initiation of these two cellular pathways was observed as early as 24 h (Fig. 3). However, only about 6% of total apoptotic cells were elicited after 72 h of incubation with 0.58 U/mL of BCA-M-PEG20 treatment and there were only about 14% total apoptotic cells even when the drug concentration was increased to 4.64 U/mL (Fig. 2). These results were concomitant with the MTT cell viability assay that showed the maximum percentage mean suppression (max.%) of BCA-M-PEG on MKN-45 cells was only about 55% even when the drug concentration was increased to 2.5 U/mL (4.3-fold of IC_50_), suggesting that BCA-M-PEG20 might induce cytostatic effect rather than cytotoxic effect on gastric cancer cell lines. The antiproliferative effect of BCA-M-PEG20 on MKN-45 cells was mainly contributed by cell cycle arrest and autophagy. Intriguingly, our BCA-M-PEG20 and other arginine-depleting enzymes could also act as cytotoxic agent on different cancer cell lines such as cervical cancer (~ 80 max.% with ~ 35% total apoptotic cells) [16], non-Hodgkin’s lymphoma (~ 80 max.% with ~ 40% total apoptotic cells) [19] and lung cancer (~ 60–70 max.% with ~ 30–50% total apoptotic cells) [18]. Therefore, arginine-depleting therapy could activate multiple cellular pathways against various types of cancer cells with a specific response.

For in vivo anti-tumor efficacy study of BCA-M-PEG20 and 5-fluorouracil (5-FU, a positive control and the first treatment of choice for advanced GC) on nude mice bearing MKN-45 tumor xenografts, BCA-M-PEG20 (250 U/mouse) and 5-FU (10 mg/kg) were administrated twice a week and once a week, respectively, and about 50% of tumor suppression resulted (Fig. 4). This result demonstrated that arginine-depleting therapy achieved by BCA-M-PEG20 was as good as by 5-FU.

In conclusion, BCA-M-PEG20 significantly inhibited the growth of ASS-positive GC cell lines in vitro, while the treatment of ADI failed to do so. Mechanistic studies demonstrated that cell cycle arrest and autophagy occurred before apoptosis in MKN-45 cells in response to arginine deprivation, suggesting that BCA-M-PEG20 was a potent cytostatic drug with minor cytotoxic effect. In vivo antitumor efficacy test also proved that BCA-M-PEG20 exhibited excellent cytostatic effect on MKN-45 tumor xenografts. As BCA-M-PEG20 activated multiple inhibitory pathways toward MKN-45, revealing that it can be a potential alternative candidate for the treatment of GC.

## Supplementary Information

Below is the link to the electronic supplementary material.

Supplementary file1 (PDF 512 KB)

## Data Availability

The datasets generated during and/or analysed during the current study are available from the corresponding author on reasonable request.
