# Supplementary material for: Mono-PEGylated thermostable Bacillus caldovelox arginase mutant (BCA-M-PEG20) induces apoptosis, autophagy, cell cycle arrest and growth inhibition in gastric cancer cells
Source: Invest New Drugs. 2022 Jul 20;40(5):895–904. doi: 10.1007/s10637-022-01265-z (PMC9395487; doi:10.1007/s10637-022-01265-z)
Supplement: Supplementary file 1 — Supplementary file1 (PDF 512 KB) [file 10637_2022_1265_MOESM1_ESM.pdf]

Mono-PEGylated Thermostable *Bacillus caldovelox* Arginase Mutant (BCA-M-PEG20) Induces Apoptosis, Autophagy, Cell Cycle Arrest and Growth Inhibition in Gastric Cancer Cells

Investigational New Drugs

Sai-Fung Chung[1], Suet-Ying Tam[1], Chi-Fai Kim[1], Hiu-Chi Chong[1], Leo Man-Yuen Lee[1] and Yun-Chung Leung[1,*]

[1]Department of Applied Biology and Chemical Technology, Lo Ka Chung Research Centre for Natural Anti-Cancer Drug Development and State Key Laboratory of Chemical Biology and Drug Discovery, The Hong Kong Polytechnic University, Hung Hom, Kowloon, Hong Kong, China

*Corresponding authors:
Yun-Chung Leung, PhD.
Tel: +852-3400 8661; fax: +852-2364 9932; email: thomas.yun-chung.leung@polyu.edu.hk

Sai-Fung Chung and Suet-Ying Tam contributed equally to this work.

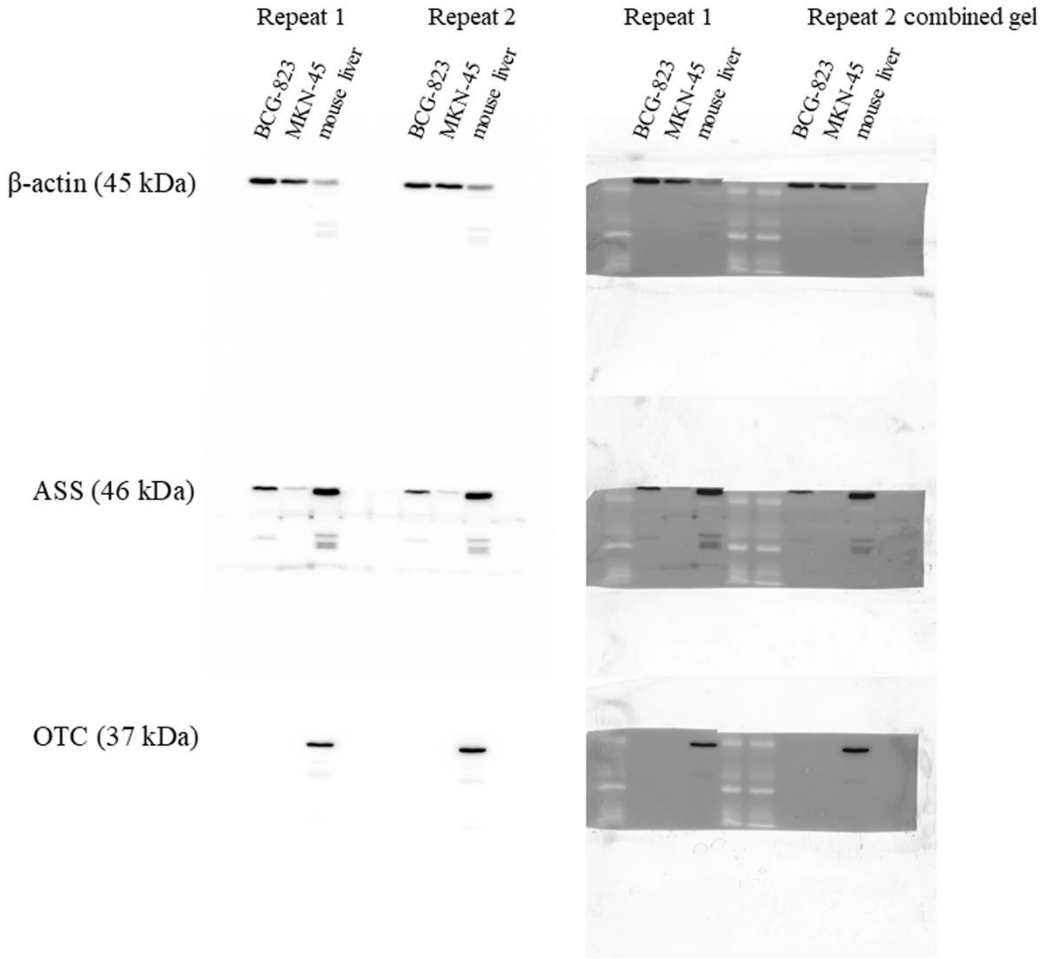

Supplementary Fig. 1 The protein expression of β-actin, ASS and OTC were measured by western blot analysis in MKN-45 and BGC-823. Mouse liver served as an OTC positive control.

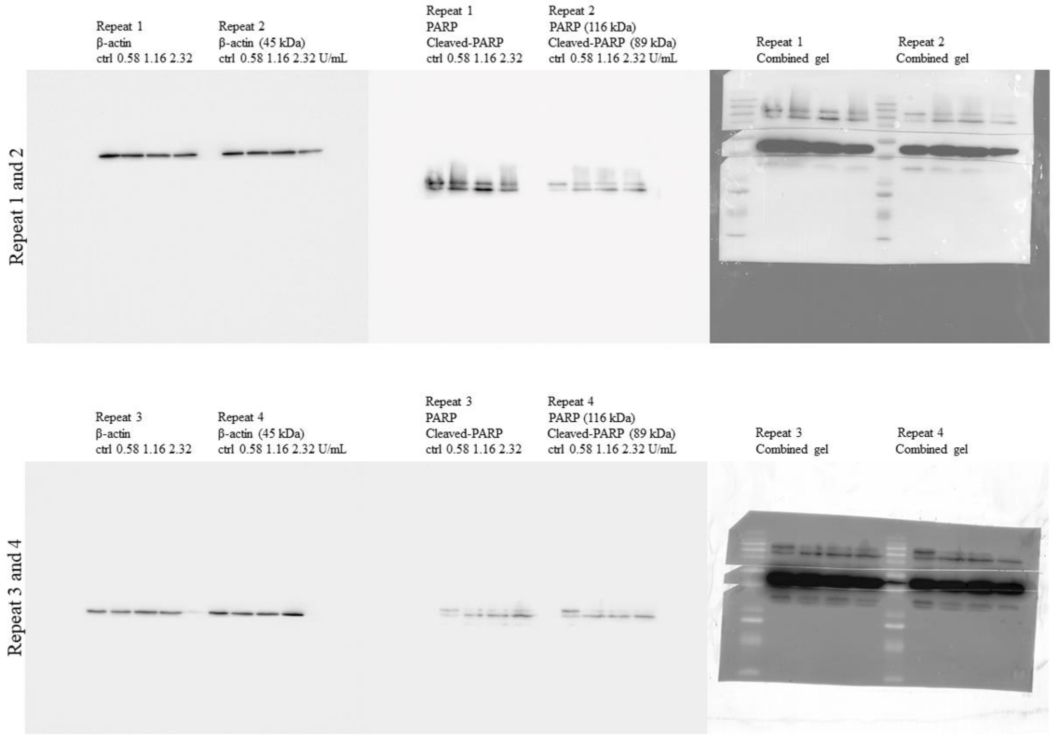

Supplementary Fig. 2 Western blot analysis of cleaved-PARP level after treatment of BCA-M-PEG20.

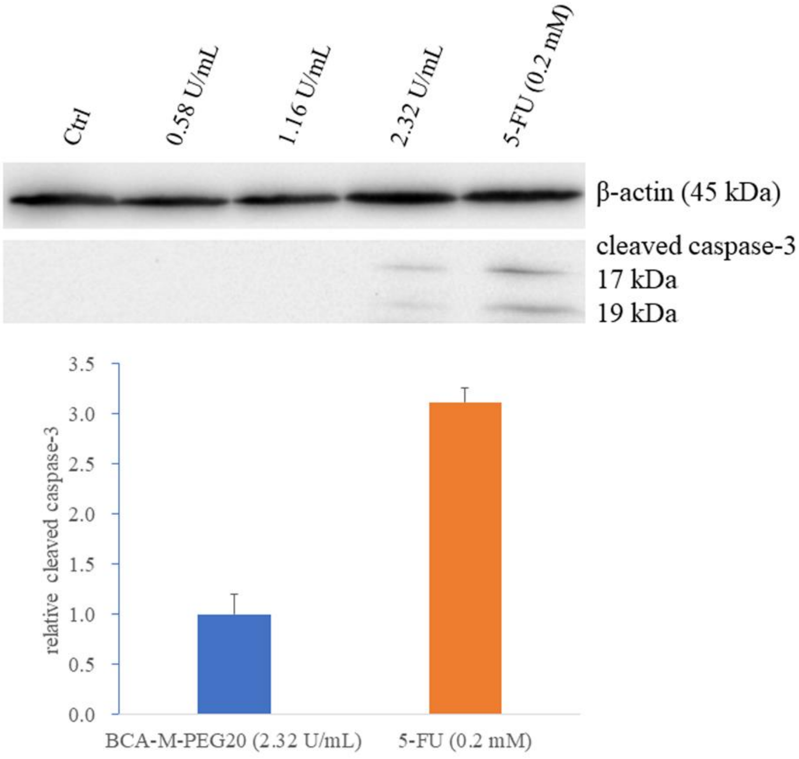

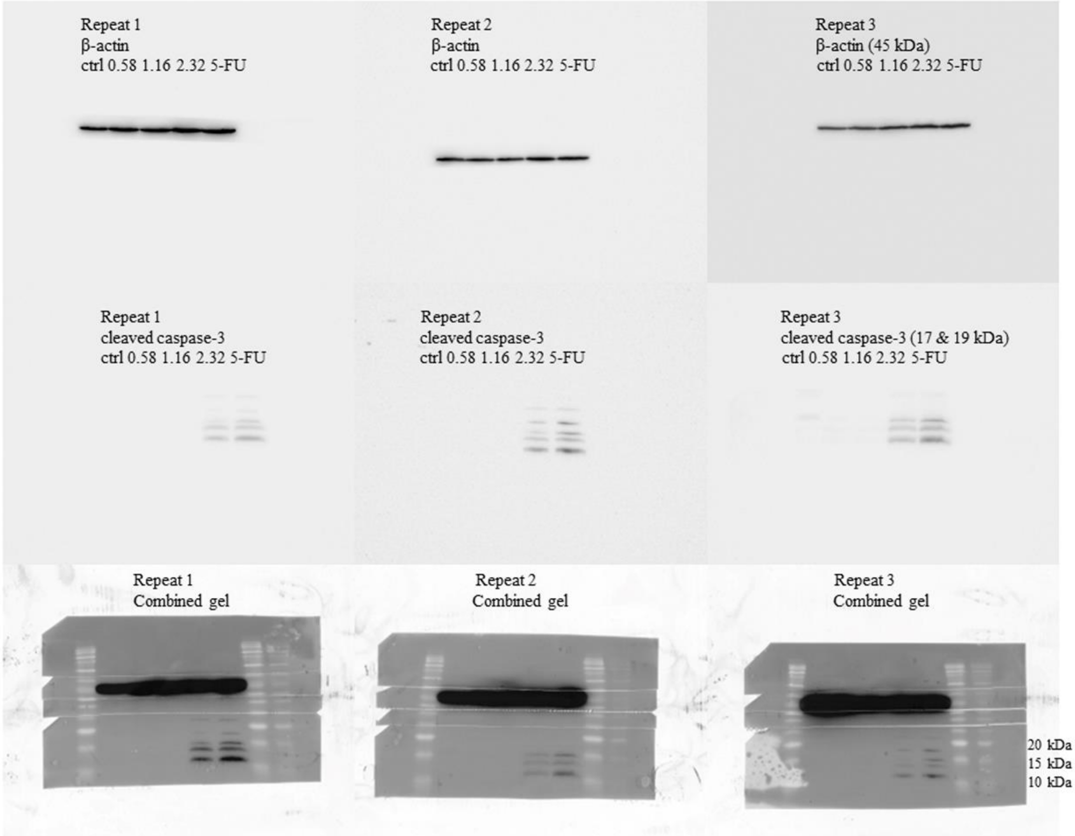

Supplementary Fig. 3 Western blot analysis of cleaved caspase-3 level after treatment of BCA-M-PEG20 and 5-FU.

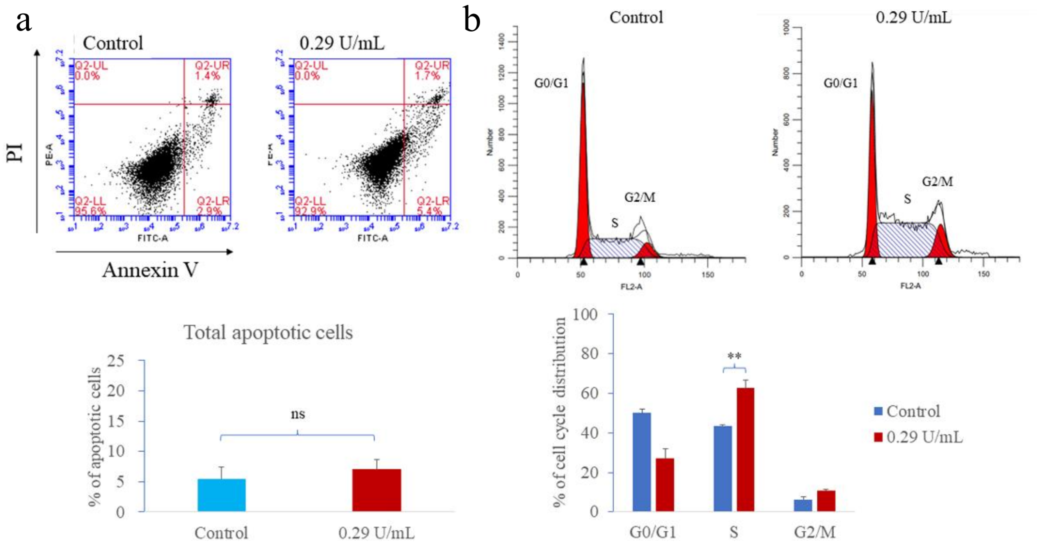

Supplementary Fig. 4 BCA-M-PEG20 with 0.29 U/mL (a) did not induce apoptosis but induced S phase arrest significantly in MKN-45 cells after 72 h of incubation.

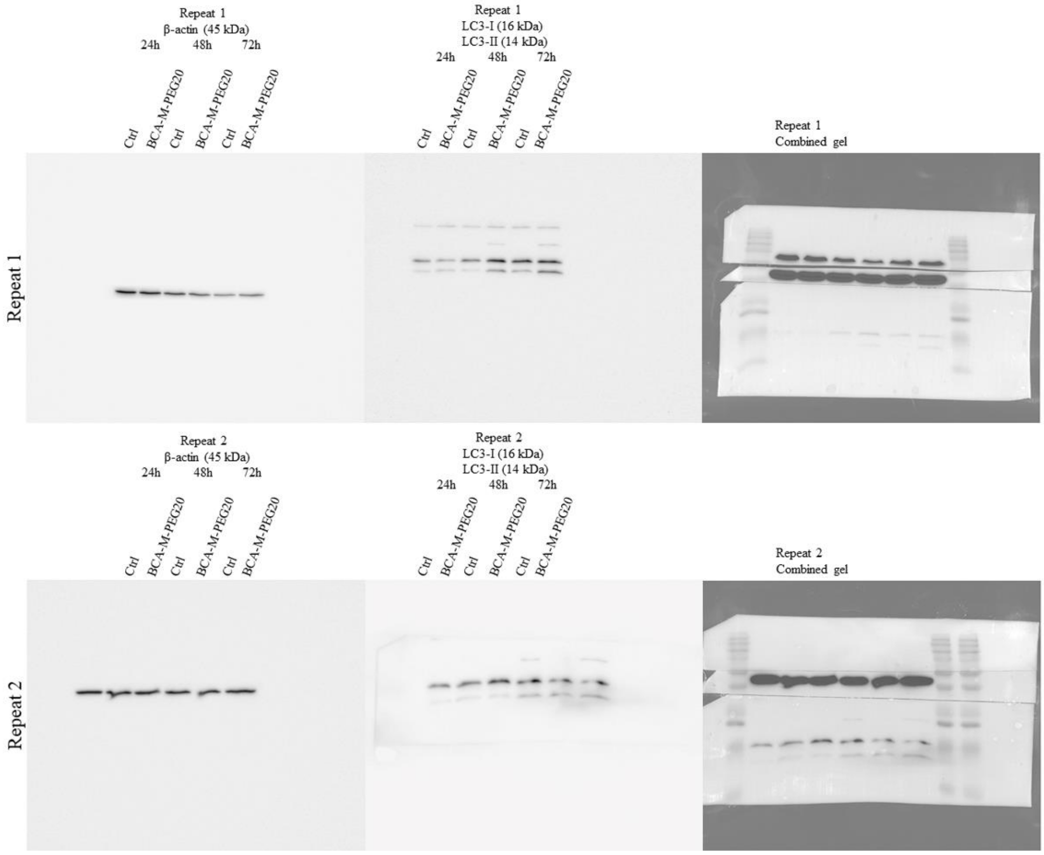

Supplementary Fig. 5 Western blot analysis of LC3-I and LC3-II level after treatment of BCA-M-PEG20.
